# Mid- and long-term functional outcomes of advancement flap for cryptoglandular perianal fistulas

**DOI:** 10.1007/s10151-025-03148-w

**Published:** 2025-05-09

**Authors:** J. Y. van Oostendorp, A. Eddarazi, C. B. H. Molenaar, D. D. E. Zimmerman, W. A. Bemelman, I. J. M. Han-Geurts

**Affiliations:** 1 Department of Surgery, Amsterdam UMC, Location AMC, Amsterdam, The Netherlands; 2 Department of Surgery, Proctos Kliniek, Bilthoven, The Netherlands; 3 Department of Surgery, Elisabeth-TweeSteden Ziekenhuis, Tilburg, The Netherlands

**Keywords:** Perianal Fistula, Advancement flap, Incontinence, Recurrence, Quality of life, Risk factors

## Abstract

**Background:**

Surgical treatment for perianal fistulas requires balancing fistula closure with the risk of complications such as incontinence. The advancement flap (AF) is a widely used sphincter-sparing technique, yet it appears to offer only marginally better outcomes compared to alternative techniques, with a notable incontinence rate. This study aimed to evaluate the success rate and long-term functional outcomes of AF at our tertiary referral center.

**Methods:**

This retrospective cohort study analyzed prospectively collected data from electronic medical records and questionnaires distributed in December 2023. Patients aged 18 or older with primary or recurrent perianal fistulas treated with AF between 2013 and 2023 were included. Fistulas of non-cryptoglandular origin and rectovaginal fistulas were excluded. The primary outcome was fecal incontinence. Secondary outcomes included disease burden, fistula closure, and risk factors for recurrence.

**Results:**

Eighty-one patients were included; 37 (46%) were women, mean age was 45 years, and 93% had a complex fistula. The median follow-up was 27 months (IQR 15.5–64). Before AF, 36% reported some degree of incontinence, increasing to 80% at long-term follow-up after AF. Specifically, 20 out of 26 (77%) preoperative fully continent patients reported incontinence issues at long-term follow-up. Fistula disease impact on daily life was higher for those who failed AF repair. Primary fistula closure was achieved in 35 patients (43%). No risk factors for AF failure could be identified.

**Conclusions:**

Advancement flap repair of perianal fistulas is challenging and can lead to fecal incontinence, so thorough preoperative counseling, consistent long-term follow-up, and further research comparing alternative sphincter-sparing techniques are warranted.

**Supplementary Information:**

The online version contains supplementary material available at 10.1007/s10151-025-03148-w.

## Introduction

Perianal fistulas are a prevalent anorectal problem, affecting 18,37 per 100,000 people in European countries [1, 2]. These fistulas pose a significant challenge due to their chronic nature and tendency for recurrence. The exact etiology of the perianal fistula remains unclear. Historically, it has been linked to an infection of the anal gland, but recent insights show that one single pathway for all perianal fistula formation seems unlikely [3, 4]. Fistulas are also related to chronic anal fissures and ongoing inflammation due to an activated immune system caused by an unknown driver. Interestingly, following drainage and debridement of an anal abscess, only 37–40% of patients develop a fistula, particularly within the first year [5, 6]. These fistulas can cause considerable morbidity, including pain, recurrent abscesses, and purulent discharge, significantly impacting the quality of life.

The current treatment of cryptoglandular perianal fistulas is based on a delicate balance between eradicating the fistula tract and preserving anal sphincter function. Preoperative workup is crucial for tailoring a surgical plan. The Parks classification system often guides the surgical approach [7]. Simple fistulas are usually best treated by lay-open fistulotomy with high success rates and a low risk of incontinence [8].

For complex fistulas, various sphincter-sparing techniques have been developed, including ligation of the intersphincteric fistula tract (LIFT), laser therapy, anal plugs, injectables, rerouting seton, and the advancement flap (AF), also known as transanal advancement flap repair (TAFR) [9]. Among these methods, AF is the most frequently used sphincter-sparing technique in The Netherlands [10]. This procedure involves excising the internal opening and advancing a flap of healthy mucosal tissue to cover the defect, thereby promoting closure and reducing the risk of recurrence.

The AF technique is valued for its sphincter-sparing properties, offering a potential solution for patients with high fistulas where sphincter preservation is paramount. However, despite the theoretical advantages of AF repair, reported outcomes vary and are influenced by multiple factors [11–13]. A comprehensive understanding of the indications, technique, functional outcomes, and risk factors for recurrence of AF is essential for optimizing patient care. Recent meta-analyses have shown that while AF healing rates are comparable to other sphincter-sparing techniques, it may have worse outcomes concerning postoperative fecal incontinence [8, 14–16].

Additionally, there is a notable gap in the literature regarding the long-term functional outcomes of AF repair, especially concerning continence preservation. Many studies lack preoperative continence status or have limited follow-up durations [14, 15]. Extended follow-up studies that integrate preoperative continence data are paramount for more definitive conclusions.

Therefore, the aim of this study was to investigate the long-term outcomes of AF surgery in our tertiary referral center. Considering that the potential for inducing incontinence, the risk of recurrence, and the disease burden of perianal fistulas are the primary considerations in treatment strategies, these outcomes were specifically examined.

## Methods

### Study design and population

We conducted a retrospective observational cohort study and used prospectively collected preoperative data from patients who underwent the AF procedure. We included patients between January 2013 and December 2023 at our tertiary referral center in The Netherlands. This study was approved by the Medical Ethics Committee of Amsterdam UMC (MEC no. 2023.0702).

### Eligibility criteria

The study included consecutive adult patients (aged 18 years and older) with primary or recurrent cryptoglandular perianal fistulas who underwent the AF procedure. Eligibility for AF covered simple fistulas and those with high tracts (upper two-thirds of the external sphincter) at risk for incontinence if treated with fistulotomy. Patients who underwent anodermal flap (house flap) procedures were excluded from this study. For anterior fistulas, AF was the primary choice for men, while LIFT was preferred for women. The decision to perform AF was always based on consensus among a group of surgeons, after considering other sphincter-preserving alternatives, and through shared decision-making with the patient. Initial management for cases with inflammation or sepsis included loose seton drainage, ensuring that patients were free from septic criteria or abscess at the time of surgery. Exclusion criteria included non-cryptoglandular fistulas, excluding those associated with Crohn’s disease, HIV, or malignancy, and rectovaginal fistulas because of their distinct recurrence patterns.

### Diagnostics

All patients underwent comprehensive physical examinations. We performed a three-dimensional endo-anal ultrasound (3D-EAUS) preoperatively to assess fistula anatomy. The proximal boundary of the anal canal was defined as the puborectal muscle, while the distal boundary was marked by the lower edge of the internal sphincter. Fistulas were categorized based on their anatomical course into intersphincteric, low, mid, or high trans-sphincteric, supra-sphincteric, or extra-sphincteric types. Specifically, “low” referred to involvement of the lower third of the external sphincter,"mid” the middle third, and “high” the upper third.

### Treatment protocol: advancement flap

All surgeries were performed by experienced colorectal surgeons specializing in perianal fistula treatment. Patients were treated in day care and received a preoperative enema the evening before and 1 h prior to leaving the house on the day of surgery to ensure a ‘clean’ rectum. Procedures were conducted under general or spinal anesthesia, with the patient’s position determined by the location of the fistula: lithotomy for posterior and prone for anterior fistulas. Preoperative antibiotic prophylaxis included intravenous cefazolin 2 g and metronidazole 500 mg. The surgical area was disinfected using chlorhexidine.

The AF procedure began with identification of the external fistula opening (EFO) and the internal fistula opening (IFO) through digital rectal examination and proctoscopy using a Czerny retractor. Absence of abscess was confirmed in all cases. The EFO was cored out until the EAS was reached, and then the rest of the fistula tract was curetted with a sharp spoon. Using a Lone Star retractor for optimal exposure, the epithelium around the IFO was excised. The size and location of the flap were determined, and submucosal infiltration with xylocaine with 2% adrenaline facilitated dissection. A flap with a broad base (2–3 cm) was meticulously dissected using diathermy, including mucosa, submucosa, and a small number of internal sphincter muscle fibers. This flap was mobilized sufficiently to cover the IFO without tension and with overlap. The IFO was closed before advancing the flap over it using Vicryl 3–0 (Ethicon Endo-Surgery, Cincinnati, OH). Hemostasis was achieved to prevent hematoma formation beneath the flap. The created flap was then sutured in the distal anal canal with interrupted Vicryl 3–0 sutures. Local infiltration with bupivacaine (Marcaine) 5 cc 5 mg/ml with adrenaline 5 mcg/ml was administered for postoperative analgesia.

Postoperative care instructions included twice-daily wound irrigation (and after defecation), maintaining soft stools with a daily sachet of macrogol, and avoiding straining or pressure on the wound. Patients were prescribed amoxicillin/​clavulanic acid 500/125 mg three times daily for 5 days, tramadol 50 mg three times daily for 7 days together with paracetamol and ibuprofen, and macrogol as needed. Follow-up was scheduled for 2 weeks post-surgery.

### Outcome measures

Our study aimed to adhere closely to AFCOS standards despite its retrospective nature [17].

#### Primary outcome measure

The primary outcome measure was fecal incontinence, assessed by the Wexner Fecal Incontinence Score (also known as the Cleveland Clinic Florida Fecal Incontinence Severity Score System). This score categorizes the severity of fecal incontinence through five questions [18, 19]. Each question is scored from 0 to 4, with the total Wexner score ranging from 0 to 20; higher scores indicate more severe fecal incontinence. A Wexner score ≥ 9 indicates a significant impairment of quality of life and is used in decision making [20]. Continence status before the AF surgery was compared to long-term follow-up responses to questionnaires, detailing incontinence types and frequencies. Sankey diagrams were employed to visualize postoperative changes in continence types [21].

#### Secondary outcome measures

Secondary outcome measures included patient-reported disease burden, assessed using the ProctoPROM score, which evaluated the fistula’s impact on daily life before and after AF surgery, as well as long-term follow-up [22]. This five-question score ranges from 0 (optimal outcome) to 50, with higher scores indicating greater disease burden.

Another key secondary outcome was fistula closure, which was assessed via clinical examination. Closure was defined as the absence of an external opening, discharge, or pain. In uncertain cases, 3D-EAUS was the primary method of assessment, performed daily by expert colorectal surgeons, providing reliable radiologic confirmation given the limited availability of MRI at our center [23]. Treatment failure was defined as persistence at 12 weeks post-surgery, and fistulas recurring after 3 months were sub-classified as recurrent.

#### Potential risk factors for AF failure

Extensive literature highlights various risk factors that influence perianal fistulas and their surgical outcomes. This study examined potential risk factors associated with AF failure, including age, gender, BMI, diabetes mellitus, smoking status, tertiary referral status, previous fistula surgery, fistula complexity, height of the fistula tract, seton drainage, location of the IFO, and surgeon experience.

### Data collection and questionnaires

Clinical data were retrospectively collected from electronic medical records, incorporating demographic details, clinical information, fistula characteristics, postoperative complications, recurrence rate, further treatment, and eventual closure rates after subsequent treatment. Information on previous fistula surgeries categorized interventions into definitive repairs versus abscess/seton drainage. Preoperative continence data and the functional impact of the fistula on daily life were systematically collected as standard protocol. Patients were routinely followed postoperatively at our outpatient clinic 2 weeks post-surgery initially and then at 4–6-week intervals up till at least 3 months or until fistula closure was confirmed.

In December 2023, comprehensive questionnaires were distributed to assess the long-term outcomes, including symptom persistence, fistula recurrence, subsequent surgeries, fecal incontinence (Wexner), and disease burden (ProctoPROM). Efforts were made to enhance response rates through follow-up communication with non-respondents via email and telephone.

### Statistical data analysis

Statistical analyses were performed using SPSS software (IBM, Armonk, NY, USA, SPSS Statistics 28). Continuous data were presented as mean values with standard deviation (SD) or median values with interquartile range (IQR), while categorical data were presented as percentages using descriptive statistics and crosstabs. Group differences were assessed using Fisher’s exact test or Fisher-Freeman-Halton exact test for categorical data, depending on distribution. Differences in continuous data were assessed with the *t*-test for parametric data or the Mann-Whitney U test for nonparametric data. Changes in scores over time within individual patients were assessed using the Wilcoxon signed rank test. Sankey diagrams were computed to visualize changes in continence status (continent vs incontinence for gas, liquid, or solid stool). Univariate hazard ratios and 95% confidence intervals were reported for fistula recurrence using Cox proportional hazard regression analyses. Kaplan-Meier survival analysis was performed to estimate fistula-free survival following AF surgery. Statistical significance was defined as *p* < 0.05.

## Results

**Fig. 1:**
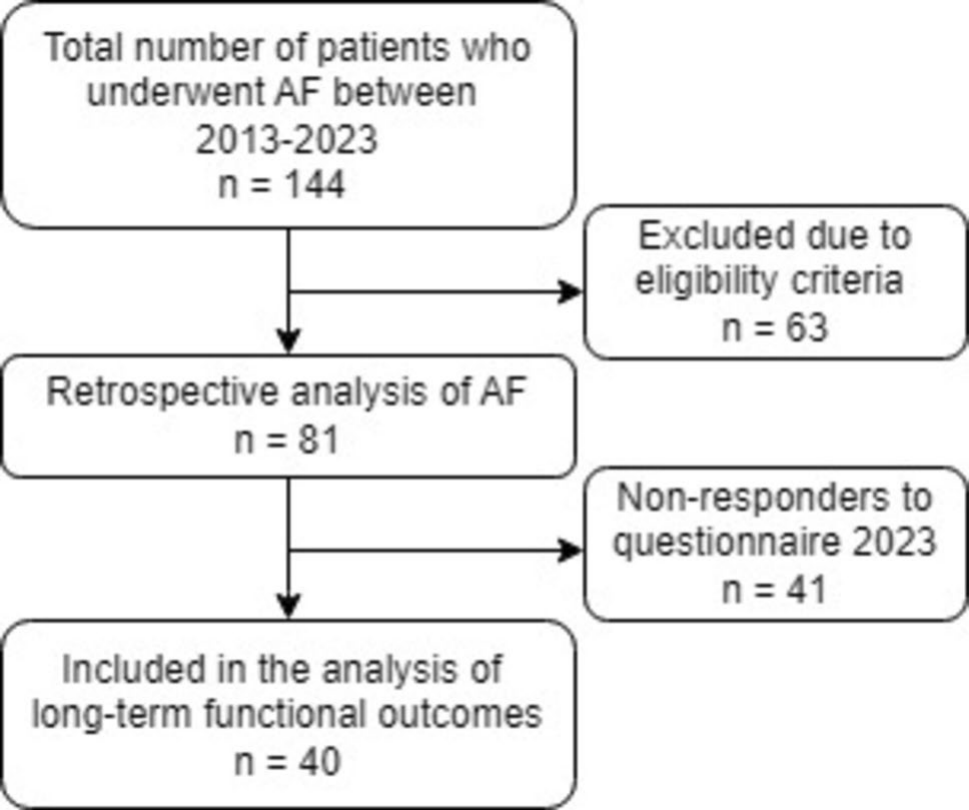
Patient flowchart

During the study period, 144 patients underwent AF repair for perianal fistulas at our center. Of these, 81 patients met the eligibility criteria and were included in the analysis (Fig. 1). The median follow-up time was 27 (IQR 15.5–64) months. Baseline characteristics are summarized in Table 1. The mean age of the cohort was 45 (SD 11) years, with females comprising 46%. The majority of patients (84%) were tertiary referrals from other hospitals because of persistent or recurrent disease. Nearly all patients (97%) had undergone previous surgery, with a median of 3 (range 0–20) surgeries. Thirty-five patients (43%) had at least one prior attempt to close the fistula, with 14 (17%) undergoing two or more attempts using various techniques. Most (74%) underwent seton drainage prior to AF surgery, with an average duration of 21 (SD 11.2) weeks. Complex fistulas constituted 93% of the cohort, with high transsphincteric fistulas accounting for over two-thirds (72%) of cases. The numbers of anterior and posterior fistulas were evenly distributed.

**Table 1:** Baseline characteristics

	Total *n* = 81
Sex, *n* (%)	
Male	44 (54)
Female	37 (46)
Age at time of surgery (years), mean (SD)	45 (11)
BMI (kg/m^2^), mean (SD)	27 (4)
Smoking, *n* (%)	24 (30)
Diabetes mellitus, *n* (%)	1 (1)
ASA I–II, *n* (%)	81 (100)
Tertiary referral, *n* (%)	68 (84)
Prior fistula surgery, *n* (%)	79 (97)
Exclusively abscess drainage or seton placement	44 (54)
Prior surgical attempts aimed at fistula repair	35 (43)
Number of total prior fistula surgeries, median (range)	3 (0–20)
Number of prior fistula repair attempts, *n* (%)	
0	46 (57)
1	20 (25)
2	10 (12)
3 or more	4 (5)
Unknown	1 (1)
Number of prior fistula repair attempts, median (range)	1 (1–5)
Types of prior fistula repair attempts, *n* (%)	
Advancement flap	10 (12)
Fistulotomy	9 (11)
Fistulectomy	2 (3)
Laser (FilAC)	7 (9)
LIFT	6 (7)
Bio-LIFT	1 (1)
Plug	3 (4)
Permacol paste	6 (7)
Closure of IFO with suture	2 (3)
Seton drainage prior to AF, *n* (%)	
Yes	60 (74)
No	21 (26)
Seton drainage uration (weeks), mean (SD)	21 (11.2)
Preoperative classification of the fistula tract (Parks), *n* (%)	
Intersphincteric	5 (6)
Transsphincteric low	1 (1)
Transsphincteric mid	12 (15)
Transsphincteric high	58 (72)
Suprasphincteric	1 (1)
Extrasphincteric	4 (5)
Complexity of the fistula tract (Parks), *n* (%)	
Simple	6 (7)
Complex	75 (93)
Location of the IFO, *n* (%)	
Anterior	23 (28)
Posterior	28 (35)
Lateral right	6 (7)
Lateral left	3 (4)
Missing	21 (26)
Location of the EFO, *n* (%)	
Anterior	27 (33)
Posterior	29 (36)
Lateral right	7 (9)
Lateral left	3 (4)
Missing	15 (18)

*SD* standard deviation, *BMI*, body mass index, *LIFT*, ligation of the intersphincteric fistula tract, *IFO* internal fistula opening, *EFO* external fistula opening

### Fecal incontinence

Data on preoperative continence status were available for all 81 included patients (Table 2). Complete baseline Wexner scores were available for 24 patients. Postoperative continence status data were available from 40 patients (50%), with a median respondent follow-up time of 27.5 (range 9–115) months. Thirty-four patients could not be contacted or did not respond to the questionnaire, and seven were unwilling to comply. There were no significant differences in patient or fistula characteristics between the respondents and non-respondents.

**Table 2:** Continence status stratified by advancement flap success

	Total	AF success	AF failure	*P* value
	*n* = 81	*n* = 35	*n* = 46	
Preoperative continence status, *n* (%)				0.64
Continent	52 (64)	21 (60)	31 (67)	
Incontinent-all:	29 (36)	14 (40)	15 (33)	
Gas	10 (12)	4 (11)	6 (13)	
Liquid stool	16 (20)	8 (23)	8 (17)	
Solid stool	3 (4)	2 (6)	1 (2)	
Preoperative Wexner scores*				
Median (IQR)	9 (7–12)	12 (9–13)	8 (6.5–10.5)	0.15

Respondents to questionnaires in 2023	Total	AF success	AF failure	*P* value
	*n* = 40	*n* = 17	*n* = 23	
Postoperative continence status, *n* (%)				0.25
Continent	8 (20)	5 (29)	3 (13)	
Incontinent – all:	32 (80)	12 (71)	20 (87)	
Gas	8 (20)	2 (12)	6 (26)	
Liquid stool	19 (48)	7 (41)	12 (52)	
Solid stool	5 (12)	3 (18)	2 (9)	
Postoperative Wexner scores				
Median (IQR)	4 (1–7)	1 (0–5.5)	4.5 (3–8)	0.04

^*^Complete preoperative Wexner scores were available for 24 patients

Preoperatively, median Wexner score was 9 (IQR 7–12). Sixty-four percent of the patients reported being fully continent, and 36% reported some degree of incontinence. Specifically, 10 patients (12%) reported incontinence for gas, 16 patients (20%) for liquid stool, and 3 (4%) for solid stool. There were no significant differences in preoperative Wexner scores when stratified by AF success or failure, with medians of 12 and 8, respectively (*p* = 0.150).

Postoperative long-term respondents revealed a median Wexner score of 4 (IQR 1–7). Eight patients (20%) reported full continence with a Wexner score of 0. Among the 32 patients (80%) who experienced some degree of incontinence, 8 (20%) reported incontinence for gas, 19 (49%) for liquid stool, and 5 (12%) for solid stool. While Wexner scores decreased in both the AF success and failure groups, the reduction was more pronounced in the AF success group (median 1 vs. 4.5; *p* = 0.039).

**Fig. 2:**
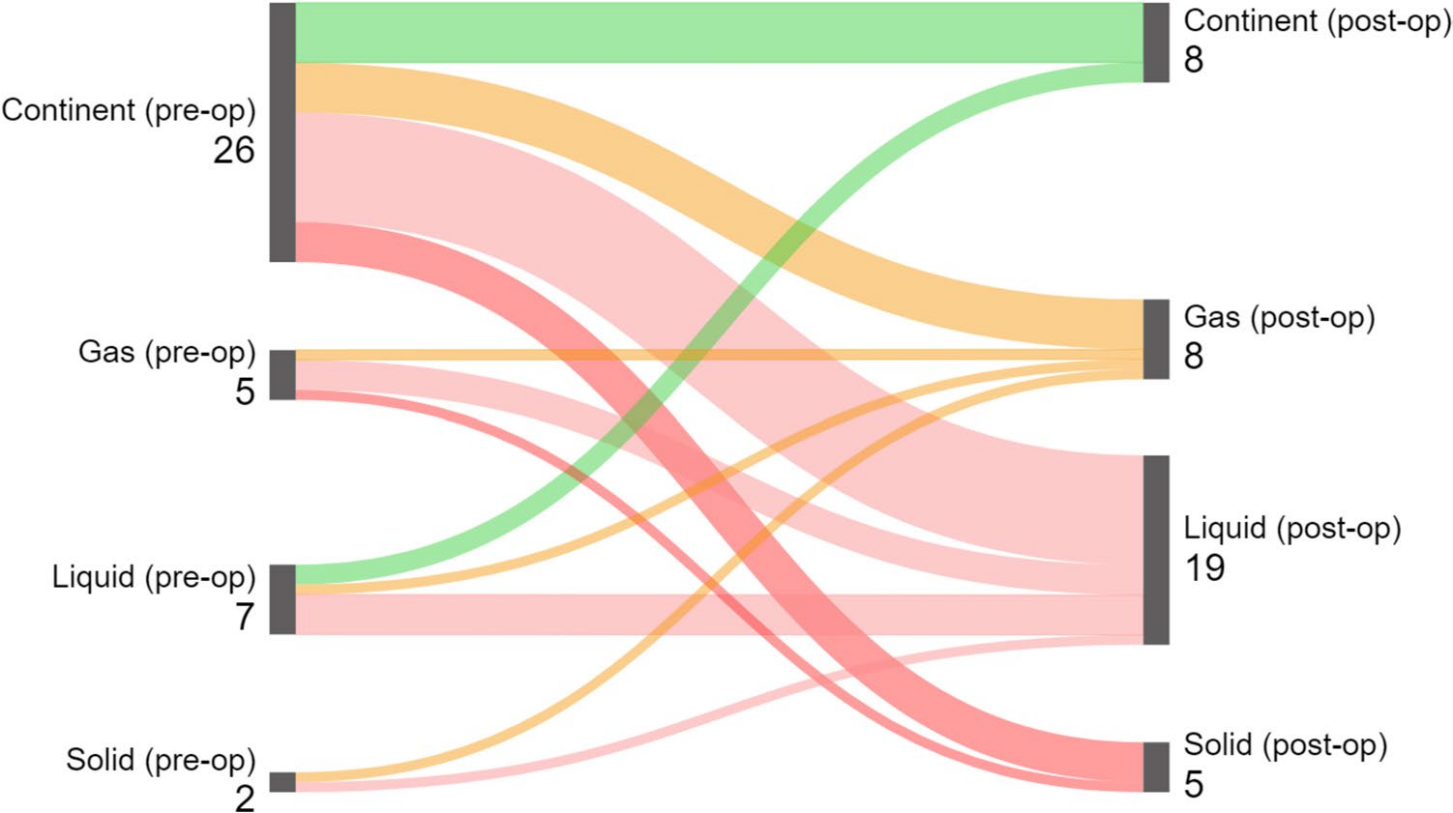
Sankey diagram of individual patient trajectories for all questionnaire respondents (*n* = 40)

However, Table 3 shows that of 26 fully continent patients preoperatively, 20 reported some degree of incontinence at long-term follow-up. Also, four patients experienced worsening: three progressed from gas to liquid stool incontinence and one to solid stool incontinence. This is best illustrated in the Sankey diagram (Fig. 2), highlighting significant long-term deterioration in continence status.

**Table 3:** Change in continence per individual case, excluding non-respondents

Respondents in 2023, *N* = 40					
Preoperative continence status, *n*	Long-term continence status (2023), *n*				
	Continent	Gas	Liquid	Solid	Total
Continent	6	**5**	**11**	**4**	26
Gas	0	1	**3**	**1**	5
Liquid	2	1	4	0	7
Solid	0	1	1	0	2
Total	8	8	19	5	40

*p* = 0.935. Bold values higlight the number of patients with worse continence status

A subset analysis of long-term Wexner questionnaire respondents with successful AF repairs (*n* = 17) showed similar results (Supplementary Table 1 and Supp Fig. 1). Of these, ten were fully continent preoperatively. Postoperatively, four (40%) reported liquid stool incontinence, two (20%) reported solid stool incontinence, and one progressed to solid stool incontinence.

### Impact on daily life

Preoperative ProctoPROM scores were available for 66 patients (81%) (Supplementary Table 2), with no significant baseline differences between the AF success and failure groups (*p* = 0.548). Short-term postoperative scores showed significant reductions in both groups (median 6.9 vs. 27). Patients with healed fistulas had significantly lower median ProctoPROM scores compared to those with unhealed fistulas (5.5 vs 12; *p* = 0.033).

Long-term assessment revealed further reductions in ProctoPROM scores for the healed fistula group (median 3, IQR 1–6). In contrast, patients with persistent or recurrent disease after AF failure reported significantly higher long-term ProctoPROM scores (median 20, IQR 4–31) compared to the healed group (median 3, IQR 1–6; *p* < 0.001).

### Fistula closure success rate

**Fig. 3:**
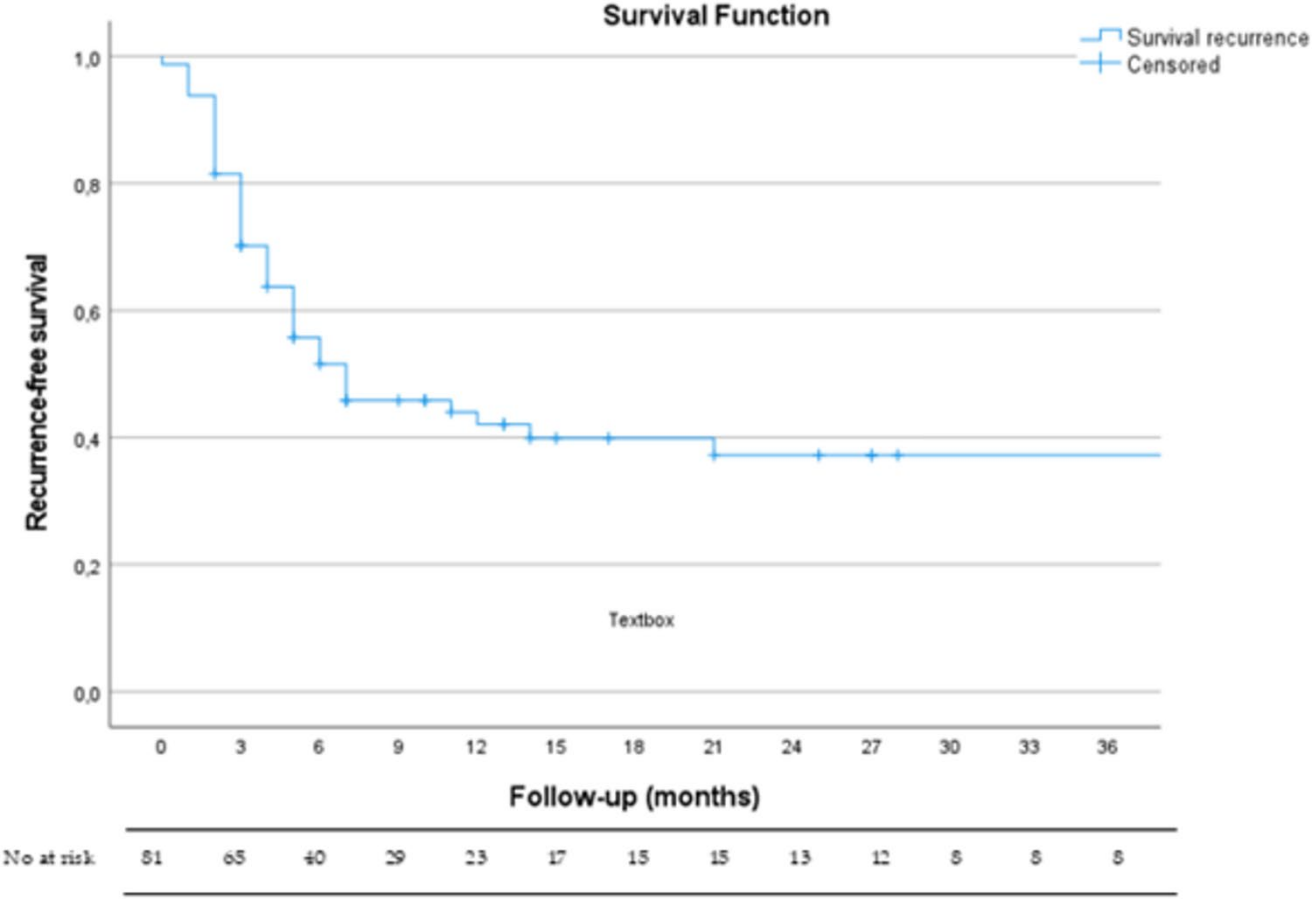
Kaplan-Meier curve recurrence-free survival after advancement flap

The success rate of AF was 43%, with 46 patients (57%) having an unhealed fistula post-AF, including 18 (22%) subclassified as recurrences. The median time to radiologic confirmation of AF failure via 3D-EAUS was 3 (IQR 2–5) months. The median recurrence-free survival was 7 (95% CI 1.26–12.74) months (Fig. 3). For patients without recurrence (or persistence), the median follow-up was 17 (95% CI 6.6–27.4) months. No additional fistula tracts were identified. Postoperative complications occurred in 11 patients (14%): eight (10%) developed infections requiring antibiotics and three (4%) experienced postoperative bleeding; all were conservatively managed.

In cases of AF failure, the median time to subsequent surgery was 6 (IQR 3–9.75) months. As of December 2023, patients with healed fistulas required an average of 1.7 (SD 1.0) surgeries, with 11 (14%) patients requiring two, 6 (7%) requiring three, and 5 (6%) needing four or more surgeries to achieve closure.

Initial management included abscess or seton drainage in 15 patients (33%). Among definitive treatments, success rates were as follows: fistulotomy 6/7 (85%), redo-AF 1/5 (20%), laser therapy 1/5 (20%), closure of IFO with sutures 1/4 (25%), Permacol paste 0/4 (0%), and LIFT 0/1 (0%). A “wait and see” approach was applied in eight patients, eventually resulting in tract closure in four (50%), suggesting that extended observation may be beneficial in select cases.

Ultimately, 16 patients were referred to other hospitals, with 12 undergoing adipose-derived stromal vascular fraction enriched with platelet-rich plasma during flap repair, achieving a 75% cure rate after a minimum follow-up of 3 months. The detailed treatment trajectories from AF surgery to the final procedure leading to fistula closure/referral are illustrated in Supplementary Fig. 2.

### Potential risk factors for AF failure

Thirteen potential risk factors for AF failure were assessed, but none reached statistical significance in the univariate Cox proportional hazard regression analysis (Supplementary Table 3), precluding a multivariate model. Briefly, one prior attempt to close the fistula was associated with an increased hazard ratio (HR) of 1.61 (95% CI 0.85–3.01; *p* = 0.147), while two or more prior surgeries showed a lower HR for failure (HR 0.62, 95% CI 0.27–1.46; *p* = 0.275). Seton drainage prior to AF was linked to a higher hazard of failure (HR 1.45, 95% CI 0.72–2.93; *p* = 0.295), although this was not statistically significant. Additionally, the surgeons’ years of experience with fistula surgery did not significantly impact the AF success rate.

## Discussion

This retrospective study, utilizing prospective data on functional outcomes, highlights the long-term efficacy and challenges associated with the AF procedure for treating perianal fistulas. Our findings provide key insights into the procedure's impact on patient outcomes and underscore important considerations for clinical practice.

Preoperative continence data showed that 64% of patients were fully continent. Postoperatively, this rate decreased disturbingly, with only 20% of respondents remaining fully continent (Wexner score 0). Among the 32 respondents reporting postoperative complaints, 48% experienced incontinence for liquid stool. This significant deterioration suggests that AF repair, although considered sphincter-sparing, adversely affects continence. This aligns with a previous study describing AF’s impact on fecal continence [24]. However, other studies report more favorable outcomes, with 80% of patients having Wexner scores of zero postoperatively and only 3% having a postoperative Wexner score > 3 [25]. This discrepancy might be explained by the number of patients in our cohort who had undergone previous surgeries, suggesting pre-existing sphincter damage. Additionally, we used the Wexner questionnaire to assess postoperative continence, although it can sometimes make it difficult to differentiate between types of incontinence, such as soiling and liquid stool, which may result in varying patient interpretations.

These functional outcomes raise concerns about the impact of AF on sphincter function, as incorporating too many internal anal sphincter (IAS) muscle fibers into the flap may compromise the functional integrity of the sphincter complex. However, a small series using 3D-EAUS to examine IAS damage post-AF found no association between the level of IAS division and continence issues [26]. Similarly, another study showed that full-thickness flaps do not increase the risk of incontinence [27], although this was contradicted by a meta-analysis [28]. High-quality prospective studies with adequate follow-up are required to clarify the long-term impact of AF on sphincter function.

Furthermore, previous studies indicate that the type of retractor used can impact functional outcomes [29, 30]. In our study, this was not a concern, as we did not use a Parks retractor. To our knowledge there is no evidence suggesting that the Lone Star retractor negatively impacts anal sphincter pressures, making it likely that the observed effects are attributable to the AF surgery itself.

To further clarify changes in sphincter function, further research should explore how mucosal resection could lead to the loss of anal symmetry, a key factor for preserving fine continence of gas and fluids. Disruption of sensory feedback and nerve supply from mucosal resection could also contribute to incontinence.

Uribe et al. reported a significant reduction in resting and squeeze pressures 3 months post-surgery, indicating that AF repair may adversely affect sphincter contractility [25]. Therefore, preserving the distal internal sphincter is crucial for maintaining adequate resting pressure and preventing anal margin deformity [31]. Advanced imaging and anorectal function tests, such as 3D high-resolution anorectal manometry (3D-HRAM), can improve candidate selection for AF, particularly in patients with prior anal surgery [25, 32]. Future research should explore the integration of additional diagnostic tools into preoperative assessments to refine patient selection.

Recent literature suggests that the LIFT procedure may offer better outcomes for continence preservation [8, 15, 33, 34]. However, meta-analyses reporting zero percent incontinence rates appear unrealistic, as our own analysis revealed a similar impact on continence status compared to AF repair [35]. Thus, further prospective studies are necessary to thoroughly evaluate the functional outcomes of alternative techniques.

ProctoPROM scores revealed high preoperative values, reflecting the significant disease burden on patients’ daily lives. Postoperatively, all patients exhibited significant reductions in ProctoPROM scores, indicating initial improvement. However, long-term follow-up showed that while the healed fistula group maintained low ProctoPROM scores, the failure group experienced a significant increase, indicating a persistent high disease burden. This underscores the importance of achieving and maintaining fistula closure for better daily functioning and quality of life.

The AF procedure had a success rate of 43% in closing the fistula, which is lower than rates reported in previous literature [8, 14, 28, 36]. This reduced success rate may be attributed to the high proportion of complex fistulas, tertiary referrals, and patients with a history of previous surgical interventions in our cohort. Consistent with existing literature, multiple surgical attempts are linked to decreased success rates in AF procedures [24, 37]. In our cohort, patients with one prior surgical closure attempt demonstrated a lower success rate compared to those without any prior attempts, although this trend did not reach statistical significance.

Despite the complexity of these cases, our results align more closely with a recent American study reporting recurrences in half of the patients undergoing sphincter-sparing procedures for complex fistulas [13]. Perhaps AF may no longer be the first choice for treating complex fistula, with alternatives like ligation of the intersphincteric fistula tract (LIFT) or adipose-derived stromal vascular fraction enriched with platelet-rich stroma cells (PRS) potentially being more appropriate [15, 38].

Extensive efforts have been made to identify risk factors for AF failure; however, results remain highly ambiguous, with no consensus on clear guidelines for either discarding or advocating AF in specific patient populations [24, 32, 39–42]. In our cohort, none of the potential risk factors for AF failure reached statistical significance, probably because of the limited sample size. The number of previous surgeries did not significantly affect AF outcomes, which contrasts with some studies but aligns with others, underscoring the variability in fistula treatment results [24, 40]. Fistula complexity (height) also did not influence the success rate, consistent with existing literature [43]. Additionally, surgeon experience did not emerge as a significant predictor, confirming that patient and fistula characteristics are more crucial determinants of outcome [44].  This suggests that within a group of dedicated proctologists, patient-specific factors play a more decisive role in treatment outcomes.

Moreover, seton placement prior to AF repair remains debated. In our cohort, the group with a seton showed more recurrences (60% vs. 48%), aligning with previous literature [39, 42, 45]. Seton placement may lead to a fibrotic tract, hindering AF success. Routine seton should be considered only in cases of abscesses or high chronic cavities but not routinely in every case, as was previously suggested by other authors [25, 46].

### Study limitations

We acknowledge several limitations in this study. First, despite multiple attempts, only 50% of patients responded to the long-term functional outcome questionnaires, with some declining participation because of the emotional burden of ongoing complaints or failed treatments. Second, the single-center, retrospective design and a cohort predominantly consisting of patients with long-standing anal complaints and referrals for recurrent disease may limit the generalizability of our findings to other healthcare systems and patient populations. Third, the lack of routine postoperative imaging at 3 months impacted the uniformity of fistula closure assessments. Finally, the absences of a systematic measurement of the flap-to-anal verge distance and routine evaluation for mucosal ectropion are notable limitations, as these factors can influence the risk of complications.

## Conclusions

Our study underscores the challenging nature of treating perianal fistulas with AF, particularly in a tertiary referral setting with complex, previously treated fistulas. The observed fistula healing rate and postoperative continence issues reveal that AF, as a sphincter-sparing technique, is functionally not as effective as previously thought. This highlights the need for thorough preoperative counseling and consistent long-term follow-up using validated questionnaires. Further prospective research is needed to compare the long-term outcomes of AF with other techniques, such as LIFT, focusing on continence preservation and quality of life.

## Supplementary Information

Below is the link to the electronic supplementary material.

Supplementary Figure 1. Sankey diagram of individual patient trajectories after successful advancement flap (*n* = 17) (PNG 85 KB)

Supplementary Figure 2. Flow diagram of additional treatments after failed advancement flap (PNG 47 KB)

Supplementary Tables (DOCX 33 KB)

## Data Availability

Study data are available on reasonable request.

## Declarations

### Preregistration

The study was not preregistered in an independent, institutional registry, as it was a retrospective study design.
