# Supplementary figures and images for: Mid- and long-term functional outcomes of advancement flap for cryptoglandular perianal fistulas
Source: Tech Coloproctol. 2025 May 9;29(1):112. doi: 10.1007/s10151-025-03148-w (PMC12064625; doi:10.1007/s10151-025-03148-w)

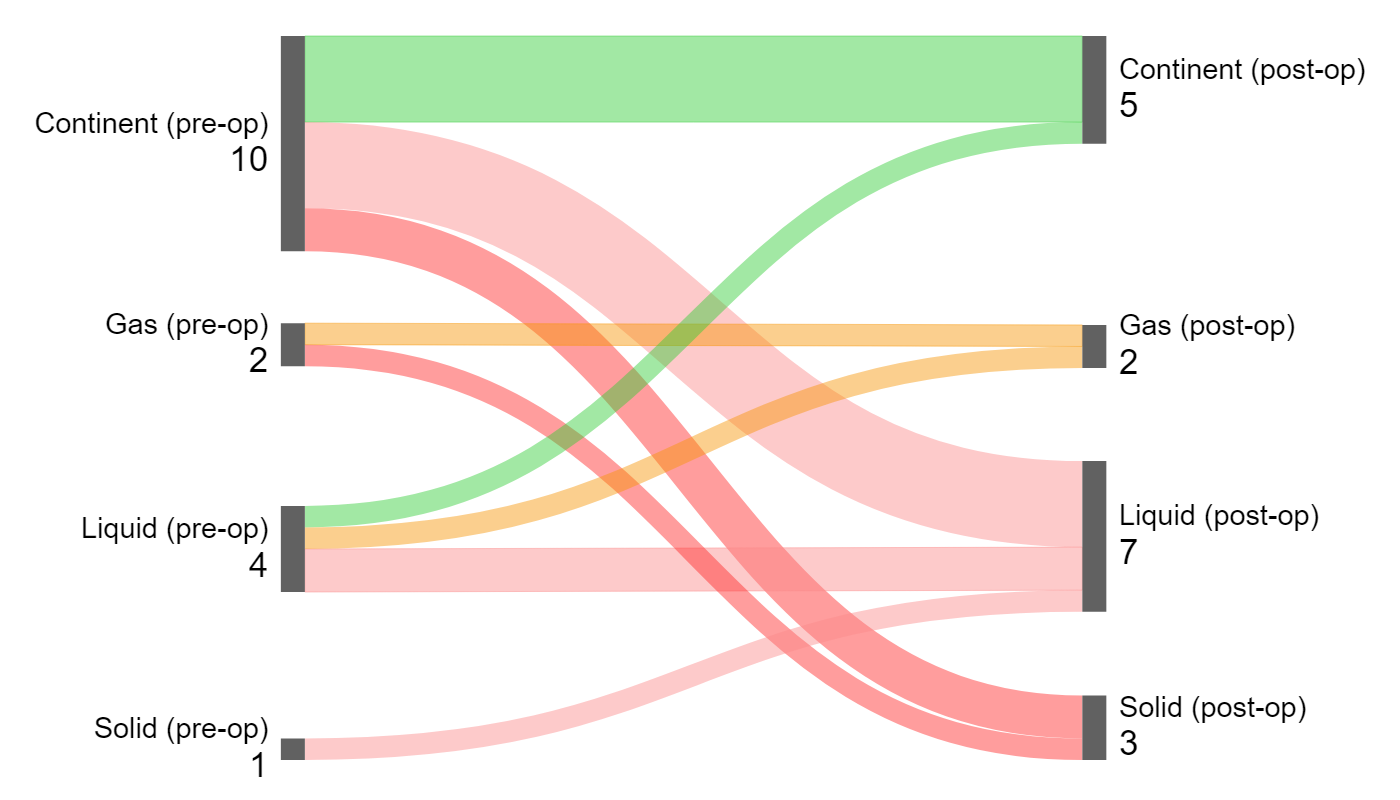

Supplement: Supplementary file 1 — Supplementary Figure 1. Sankey diagram of individual patient trajectories after successful advancement flap (n = 17) (PNG 85 KB) [file 10151_2025_3148_MOESM1_ESM.png]

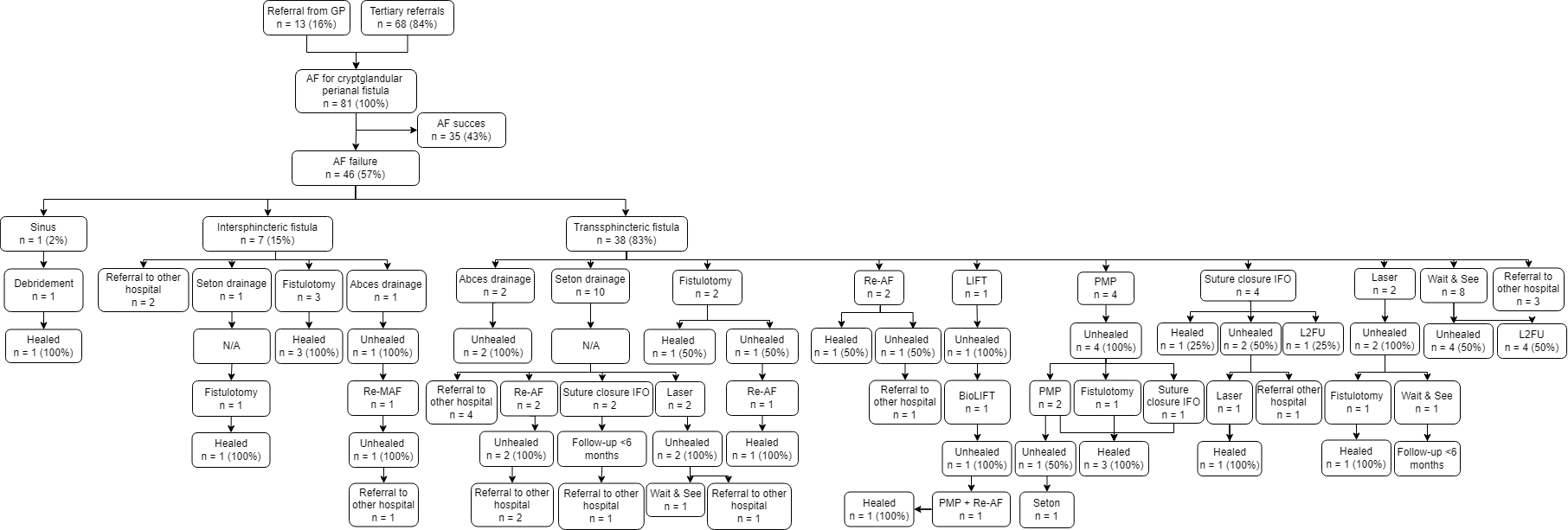

Supplement: Supplementary file 2 — Supplementary Figure 2. Flow diagram of additional treatments after failed advancement flap (PNG 47 KB) [file 10151_2025_3148_MOESM2_ESM.png]
