# Supplementary material for: Mid- and long-term functional outcomes of advancement flap for cryptoglandular perianal fistulas
Source: Tech Coloproctol. 2025 May 9;29(1):112. doi: 10.1007/s10151-025-03148-w (PMC12064625; doi:10.1007/s10151-025-03148-w)
Supplement: Supplementary file 3 — Supplementary Tables (DOCX 33 KB) [file 10151_2025_3148_MOESM3_ESM.docx]

**Appendix - Supplementary Tables**

**Supplementary Table 1.** Change in continence per individual case after successful AF repair; excluding non-respondents

| **Respondents in 2023, N = 17** | | | | | |
| --- | --- | --- | --- | --- | --- |
|  | **Long-term continence status (2023), *n*** | | | | |
| **Preoperative continence status, n** | Continent | Gas | Liquid | Solid | Total |
| Continent | 4 | 0 | **4** | **2** | 10 |
| Gas | 0 | 1 | 0 | **1** | 2 |
| Liquid | 1 | 1 | 2 | 0 | 4 |
| Solid | 0 | 0 | 1 | 0 | 1 |
| Total | 5 | 2 | 7 | 3 | 17 |

p = 0.392. Bold values highlight the number of patients with a deterioration in continence status.

**Supplementary Table 2.** ProctoPROM scores stratified by success of the Advancement Flap

| **Pre-operative ProctoPROM** | **Total**  **n = 66*** | **AF success**  **n = 29** | **AF failure**  **n = 37** | **P-value** |
| --- | --- | --- | --- | --- |
| **Total score**  Mean (SD)  Median (IQR) | 25.9 (12.6)  27 (16.9-36.2) | 25.1 (11.5)  26.5 (17-30) | 26.5 (13.5)  27.4 (14.1-37.7) | 0.548 |
| **Subcategories, median (IQR)**  Daily activities  Toilet visit  Social activities  Relationship/intimacy  Concern/worry | 5.2 (3.1-8.0)  3.0 (1.0-7.0)  5.7 (3.4-8.5)  7.0 (3.5-8.8)  6.3 (4.1-8.6) | 4.8 (3.0-7.8)  2.8 (1.2-6.5)  5.1 (3.5-8.5)  5.6 (3.1-8.3)  5.6 (2.7-9.0) | 5.9 (3.4-8.2)  3.3 (0.4-7.3)  6.6 (3.4-8.7)  8.0 (4.2-9.4)  7.3 (4.4-8.3) | 0.333  0.782  0.852  0.184  0.729 |
| **Postoperative short-term ProctoPROM** | **Total**  **n = 28** | **AF success**  **n = 16** | **AF failure**  **n = 12** | **P-value** |
| **Total score**  Mean (SD)  Median (IQR) | 10.9 (10.7)  6.9 (4-13) | 6.5 (5.3)  5.5 (2.25-10) | 16.7 (13.4)  12 (5.5-29) | **0.033** |
| **Subcategories, median (IQR)**  Daily activities  Toilet visit  Social activities  Relationship/intimacy  Concern/worry | 1 (0-3.5)  1 (0-2.75)  1 (0-2)  1 (0-3)  1 (1-5) | 1 (0-3)  1 (0-2)  1 (0-2)  0 (0-2.5)  2 (0.5-4) | 2 (0-6)  1.5 (0-6)  1.5 (0-4.7)  1.5 (0-6.2)  5.5 (3.2-6.2) | 0.807  0.508  0.446  0.186  **0.018** |
| **Postoperative long-term ProctoPROM** | **Total**  **n = 40#** | **AF success**  **n = 17** | **AF failure**  **n = 23** | **P-value** |
| **Total score**  Mean (SD)  Median (IQR) | 12.6 (12.4)  6 (2-21) | 4.5 (4.9)  3 (1-6) | 18.5 (13.0)  20 (4-31) | **<0.001** |
| **Subcategories, median (IQR)**  Daily activities  Toilet visit  Social activities  Relationship/intimacy  Concern/worry | 1 (0-4)  1 (0-5)  0.25 (0-4)  0 (0-6)  2 (0-5) | 0 (0-1.7)  1 (0-2.7)  0 (0-0.7) 0 (0-0) 1 (0-2.5) | 2 (0-5)  3 (0-5)  3 (0-7)  4 (0-7)  5 (0.7-7) | **0.023**  0.153  **0.006**  **<0.001**  **0.007** |

* Pre-operative ProctoPROM scores were retrieved for 66 out of 81 patients (81%).

# Respondents to the questionnaires in 2023. Bold p-values are below the significance threshold of 0.05.

**Supplementary Table 3.** Overview of tested risk factors for Advancement Flap failure

|  | **AF failure, n (%)** | **HR (95% CI)*** | **P-value*** |
| --- | --- | --- | --- |
| **Sex**  Female  Male | 20 (54)  26 (59) | 0.95 (0.53 – 1.71) | 0.875 |
| **Age**  ≥35 vs <35 years old  ≥40 vs <40 years old  ≥50 vs < 50 years old | 38 (60) vs 8 (44)  34 (61) vs 12 (48)  17 (57) vs 29 (57) | 1.40 (0.66 – 3.02)  1.17 (0.60 – 2.25)  0.87 (0.48 – 1.59) | 0.380  0.651  0.661 |
| **BMI**  ≥25 vs <25  ≥30 vs <30 | 26 (62) vs 11 (46)  11 (61) vs 26 (54) | 1.11 (0.55 – 2.25)  0.96 (0.48 – 1.95) | 0.771  0.918 |
| **Tertiary referral**  Yes  No | 39 (57)  7 (54) | 1.08 (0.48 – 2.41) | 0.856 |
| **Smoking**  Yes  No | 13 (54)  33 (58) | 0.83 (0.44 – 1.58) | 0.572 |
| **Total number of prior surgeries**  0-1 surgery  2 surgeries  3 or more surgeries | 10 (48)  5 (46)  31 (63) | 0.84 (0.29 – 2.45)¥  1.23 (0.60 – 2.51)¥ | 0.747  0.568 |
| **Prior fistula repair attempts**  Yes  No | 22 (63)  24 (52) | 1.08 (0.60 – 1.93) | 0.798 |
| **Number of prior fistula repair attempts**  0 surgeries  1 surgery  2 or more surgeries | 24 (52)  15 (75)  7 (47) | 1.61 (0.85 – 3.01)#  0.62 (0.27 – 1.46)# | 0.147  0.275 |
| **Complexity of the fistula**  Simple fistula  Complex fistula | 3 (50)  43 (57) | 1.06 (0.33 – 3.42) | 0.927 |
| **Hight fistula tract**  Low/mid  High | 9 (69)  32 (55) | 0.50 (0.24 – 1.07) | 0.074 |
| **Seton drainage prior to AF**  Yes  No | 36 (60)  10 (48) | 1.45 (0.72 – 2.93) | 0.295 |
| **Location of the IFO**  Anterior  Posterior  Lateral left or right | 15 (65)  14 (50)  5 (56) | 0.72 (0.35 – 1.49)$  0.71 (0.26 – 1.98)$ | 0.371  0.517 |
| **Colorectal surgeon experience**  **<**10 years  >10 years | 21 (54)  25 (60) | 0.84 (0.47 – 1.50) | 0.556 |

* Univariate Cox proportional hazards regression model. HR = Hazards Ratio; CI = confidence interval; BMI = Body mass index; IFO = internal fistula opening. ¥ Reference category = 0-1 prior surgery (i.e. drainage or seton)
# Reference category = 0 prior fistula repair attempts. $ Reference category = anterior location of the internal fistula opening.
